# Comparative Analysis of Supersonic Flow in Atmospheric and Low Pressure in the Region of Shock Waves Creation for Electron Microscopy

**DOI:** 10.3390/s23249765

**Published:** 2023-12-11

**Authors:** Pavla Šabacká, Jiří Maxa, Robert Bayer, Tomáš Binar, Petr Bača, Petra Dostalová, Martin Mačák, Pavel Čudek

**Affiliations:** 1 Faculty of Electrical Engineering and Communication, Brno University of Technology, Technická 10, 616 00 Brno, Czech Republicrobert.bayer@vut.cz (R.B.); binar@vutbr.cz (T.B.); baca@vut.cz (P.B.); 186111@vut.cz (P.D.);; 2 Institute of Scientific Instruments of the CAS, Královopolská 147, 612 64 Brno, Czech Republic

**Keywords:** Ansys Fluent, ESEM, critical flow, one-dimensional flow theory, nozzle, pressure sensors, temperature sensors, sensing techniques for low pressures

## Abstract

This paper presents mathematical-physics analyses in the field of the influence of inserted sensors on the supersonic flow behind the nozzle. It evaluates differences in the flow in the area of atmospheric pressure and low pressure on the boundary of continuum mechanics. To analyze the formation of detached and conical shock waves and their distinct characteristics in atmospheric pressure and low pressure on the boundary of continuum mechanics, we conduct comparative analyses using two types of inserted sensors: flat end and tip. These analyses were performed in two variants, considering pressure ratios of 10:1 both in front of and behind the nozzle. The first variant involved using atmospheric pressure in the chamber in front of the nozzle. The second type of analysis was conducted with a pressure of 10,000 Pa in front of the nozzle. While this represents a low pressure at the boundary of continuum mechanics, it remains above the critical limit of 113 Pa. This deliberate choice was made as it falls within the team’s research focus on low-pressure regions. Although it is situated at the boundary of continuum mechanics, it is intentionally within a pressure range where the viscosity values are not yet dependent on pressure. In these variants, the nature of the flow was investigated concerning the ratio of inertial and viscous flow forces under atmospheric pressure conditions, and it was compared with flow conditions at low pressure. In the low-pressure scenario, the ratio of inertial and viscous flow forces led to a significant reduction in the value of inertial forces. The results showed an altered flow character, characterized by a reduced tendency for the formation of cross-oblique shockwaves within the nozzle itself and the emergence of shockwaves with increased thickness. This increased thickness is attributed to viscous forces inhibiting the thickening of the shockwave itself. This altered flow character may have implications, such as influencing temperature sensing with a tipped sensor. The shockwave area may form in a very confined space in front of the tip, potentially impacting the results. Additionally, due to reduced inertial forces, the cone shock wave’s angle is a few degrees larger than theoretical predictions, and there is no tilting due to lower inertial forces. These analyses serve as the basis for upcoming experiments in the experimental chamber designed specifically for investigations in the given region of low pressures at the boundary of continuum mechanics. The objective, in combination with mathematical-physics analyses, is to determine changes within this region of the continuum mechanics boundary where inertial forces are markedly lower than in the atmosphere but remain under the influence of unreduced viscosity.

## 1. Introduction

Temperature sensing in supersonic flow using sensors presents challenges not only due to the compressibility of the gas but also because of the formation of shockwaves, which strongly affect the values of state quantities [1,2,3]. In the free flow, their value is completely different than in the flow with the inserted probe. The paper deals with the sensing of static temperature in a supersonic gas flow. There are two ways to handle this problem.

**Figure 1:**
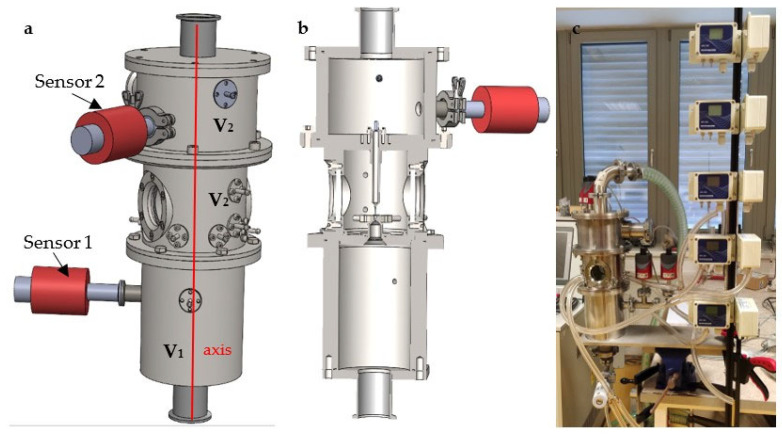
Experimental chamber, (**a**) 3D model, (**b**) cross-section of 3D model, and (**c**) real experimental chamber.

The first option is to insert a probe with a flat end into the stream, in front of which a perpendicular torn-off shock wave is created. At this point, we no longer sense the state quantities of static pressure and static temperature on the probe head; instead, we sense total pressure and stagnation temperature [4,5]. Using the stagnation temperature value, we can then calculate the static temperature. However, this method requires knowing the Mach number at the location of temperature sensing, which can sometimes be challenging. This leads to the necessity of performing velocity sensing at a specific location. One way to achieve this is by using a sensor based on a Pitot tube, which senses total pressure. However, capturing static pressure from the side of the tube is also necessary (Figure 1). This might pose a challenge in confined spaces.

The second option is a suitably shaped temperature sensor fitted with a tip corresponding to the given flow so that a cone shock wave is created on the front of the sensor, beyond which the velocity of the flow does not decrease to subsonic speed and there are no step changes in state variables [6]. In addition, these changes occur behind the tip of the sensor, not in front of it.

In this paper, a mathematical-physics analysis [7,8,9] of a temperature sensor inserted into a controlled supersonic flow generated behind the nozzle is carried out and both variants are evaluated.

These analyses were performed as a comparison of the issue in both atmospheric pressure and the low-pressure region at the boundary of continuum mechanics [10,11]. The effect of the changed ratio of inertial and viscous forces due to low pressure was evaluated, but still in the region where low pressure does not affect the viscosity value.

The research on supersonic flow at low pressures and its different characteristics of flow at low pressures on the boundary of continuum mechanics compared to conventional atmospheric pressure has had a significant impact on the development of the field of Environmental Scanning Electron Microscopy (ESEM) [12,13,14]. In general, electron microscopy has brought the possibility of viewing samples at a magnification that is several times more detailed than conventional optical microscopes [15,16]. However, electron microscopy necessitates a vacuum for the passage of the electron beam. In this environment, monitoring wet samples is challenging and requires extensive preparation [17]. As a solution, the Environmental Scanning Electron Microscope (ESEM) was developed. In this microscope, the specimen chamber is separated from the vacuum spaces by a system of small apertures and an intermediate chamber. Some devices, such as mass spectrometers, include intermediate chambers. This design allows samples to be held in the specimen chamber at pressures on the boundary of continuum mechanics. Consequently, wet samples can be observed without special preparation, and it becomes possible to study electrically non-conductive, semiconducting samples [18], or native samples [19] without damaging them, or studying these samples in dynamic in-situ experiments [20,21]. ESEM detects signal electrons using specialized ionization or scintillation detectors [22,23].

ESEM consists of chambers with a large pressure gradient separated by a small aperture, in which a critical flow is generated, which has a great impact on the scattering of the electron beam. This paper also contributes to the research on critical flow at the boundary of continuum mechanics in the field of shockwaves.

## 2. Experimental Chamber

**Figure 2:**
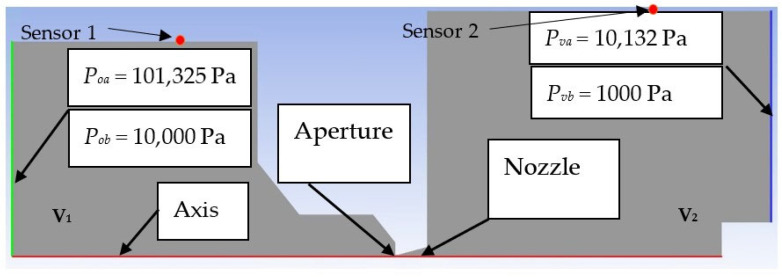
The 2D axisymmetric model of the experimental chamber with boundary conditions rotated by 90°.

These analyses serve as preparatory materials for experiments conducted in the experimental chamber, specifically designed for the comprehensive study of gas flow in supersonic mode. The chamber accommodates investigations under classical atmospheric conditions as well as in the low-pressure region at the boundary of continuum mechanics and the slip flow region (Figure 2) [24]. The chamber is comprised of two chambers separated by a replaceable component, which can be used to separate the chambers with different aperture and nozzle variants according to the type of research currently planned [25]. In the paper, a variant is analyzed where the chambers are separated by the aperture with a diameter of 1.6 mm and a subsequent nozzle with dimensions that are further determined in the paper according to the theory for the calculated cross-section. The experimental chamber was lent to Brno University of Technology by the team of Vílém Neděla from the Institute of Scientific Instruments of the Czech Academy of Sciences, who manufactured it and are engaged in research in the field of low-pressure flow, among other things. The results of the research in the field of the difference in the character of supersonic flow at low pressures will be used in the construction of a differentially pumped chamber in ESEM. In this conclusion, shock waves are analyzed, which, since there are pressure gradients on them, have a great influence on the primary electron beam that passes through the differentially pumped chamber because there is a greater scattering of the electron beam on them. Each scattering has the effect of reducing the resulting sharpness of the image.

A 2D axisymmetric model of the chamber with aperture and nozzle was created for further theoretical calculations and mathematical-physics analyses. As mentioned, the aperture diameter was chosen to be 1.6 mm.

Analyses were performed for two pressure drops. The first one is the atmospheric pressure variant (*P_o_* = 101,325 Pa and output pressure *P_v_* = 10,132 Pa, i.e., a ratio of 10:1). From the given ratio, the calculation dimension of the nozzle was further determined using the theory of one-dimensional isentropic flow (see below). Boundary conditions for the analyses are shown in Figure 2. Subsequently, for the same pressure ratio, an analysis was performed for the low-pressure variant (*P_o_* = 10,000 Pa and output pressure *P_v_* = 1000 Pa).

This second option is chosen because all our research is in the low-pressure area but still in the area of continuum mechanics. In this case, with a choice above 133 Pa, the viscosity of the gas is not dependent on the pressure applied. The derivation of the dynamic viscosity relationship concerning the mean free path (Equation (1)) was taken from [26].

(1) $$\eta =\frac{1}{4\pi r^{2}}\sqrt{\frac{m_{0}kT}{\pi }}$$

This relationship leads to the surprising conclusion that dynamic viscosity does not depend on the pressure and density of the gas. Physically, it can be justified by the fact that at a lower density of the gas, fewer molecules jump between layers, but due to the longer free path, each jump is associated with a proportionately greater momentum transfer. Experiments have confirmed this conclusion for gases under conditions under which a gas can be considered ideal.

Pfeiffer probes were used as sensors that sensed the absolute pressure in the chambers. Their location can be seen in Figure 1, left; these are red probes inserted into the appropriate chamber. Their location is also shown in the diagram in Figure 2. According to the current measurements, the Pfeiffer CMR 361 sensors with a measuring range from 10 Pa to 110,000 Pa and the Pfeiffer CMR 362 sensor with a measuring range from 1 Pa to 1100 Pa are used.

## 3. Methodology

As a first step, using the theory of one-dimensional isentropic flow, the calculated state of the nozzle for the selected angle of 12° was determined for the selected ratio of *P_v_/P_o_* = 0.1 [27,28]. Subsequently, an analysis of this nozzle was performed on the previously tuned Ansys Fluent system [24], and the results were evaluated regarding the critical flow theory. This combination of theory and mathematical-physics analyses is one of the great advantages of modern research methodology [29,30,31].

Subsequently, analyses of supersonic flow in the nozzle and behind the nozzle are carried out in three variants for both of the above-mentioned pressure conditions.

- Flow in free space, in an intact environment—Free Flow.
- Flow in an environment with an inserted temperature sensor with a flat end—Flat shape.
- Flow in an environment with an inserted temperature sensor with a conical end—Angle 30°.

These analyses require theoretical knowledge in the given areas, which will now be briefly introduced:

Understanding the three regimes of gas flow:

- Incompressible regime
- Compressible subsonic regime
- Compressible supersonic regime

The last point is also related to the change in temperature behind the perpendicular shock wave.

Another consideration is the behavior of the compressible supersonic flow mode at the tip of the sensor, on which not a detached perpendicular shock wave but a cone shock wave is created.

In the end, the whole research is carried out using a modern methodology, combining these physical theories with mathematical-physics analyses using the Ansys Fluent system, preparing experimental measurements using sensors, and thus verifying the results and retrospectively tuning the mathematical-physics analysis using the Ansys Fluent system. The results of experimental measurements will thus be harmonized with mathematical-physics analysis [32,33].

### Simulation Settings in the Ansys Fluent System

For this type of analysis, the Pressure-Based Coupled solver has proven to be less computationally demanding after comparative analyses and, at the same time, delivers the same results as the Density-Based solver.

**Figure 3:**
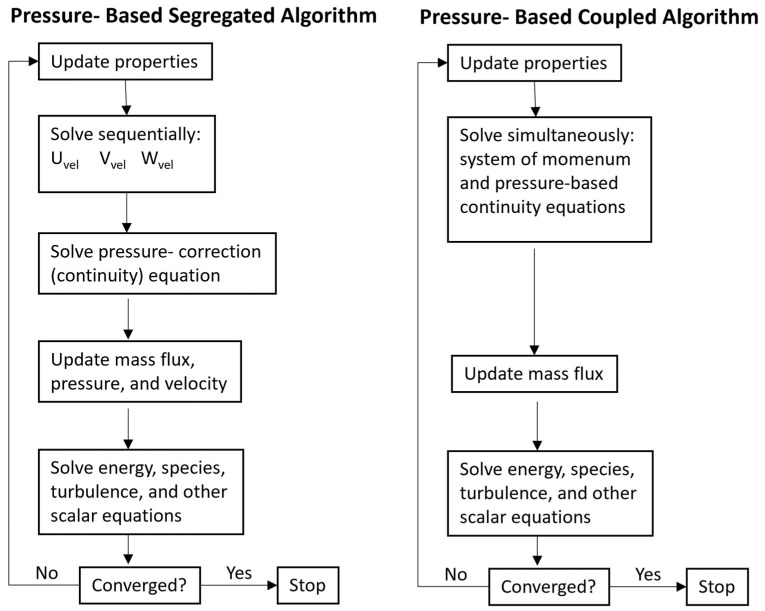
Overview of the Pressure-Based Solution Methods.

It is a Pressure-based solver that employs an algorithm that belongs to a general class of methods called the projection method [34]. In the projection method, the limitation of conservation of mass (continuity) of the velocity field is achieved by solving the pressure equation (or by correcting the pressure). Unlike the segregated algorithm described above, its scheme and comparison with the coupled variant are shown in Figure 3 [35], and the pressure-based coupled algorithm solves a coupled system of equations comprising the momentum equations and the pressure-based continuity equation. Thus, in the coupled algorithm, Steps 2 and 3 in the segregated solution algorithm are replaced by a single step in which the coupled system of equations is solved. The remaining equations are solved in a decoupled fashion, as in the segregated algorithm. Since the momentum and continuity equations are solved in a closely coupled manner, the rate of solution convergence significantly improves when compared to the segregated algorithm. However, the memory requirement increases by 1.5–2 times that of the segregated algorithm since the discrete system of all momentum and pressure-based continuity equations needs to be stored in the memory when solving for the velocity and pressure fields (rather than just a single equation, as is the case with the segregated algorithm).

The solution process involves iterations in which the entire set of governing equations is repeatedly solved until the solution converges since the governing equations are nonlinear and connected [36].

In the next setting, the Advection Upstream Splitting Method (AUSM) scheme was selected. It is a numerical method used for solving advection equations in computational fluid dynamics. It is particularly useful for the simulation of compressible flows with shocks and discontinuities, which fully corresponds to our case of solving large gradients associated with shock waves. The AUSM is developed as a numerical inviscid flux function for solving a general system of conservation equations. It is based on the upwind concept and was motivated to provide an alternative approach to other upwind methods, such as the Godunov method, flux difference splitting methods by Roe, and Solomon and Osher, and flux vector splitting methods by Van Leer, and Steger and Warming. The AUSM scheme first computes a cell interface Mach number based on the characteristic speeds of the neighboring cells. The interface Mach number is then used to determine the upwind extrapolation for the convection part of the inviscid fluxes. A separate Mach number splitting is used for the pressure terms. Generalized Mach number-based convection and pressure splitting functions were proposed by Liou [35] and the new scheme was termed AUSM+. The AUSM+ scheme has several desirable properties:

1. Provides exact resolution of contact and shock discontinuities
2. Preserves positivity of scalar quantities
3. Free of oscillations at stationary and moving shocks

To solve the transfer of results between the cell’s mesh, the second-order upwind scheme was chosen, where variables on cell surfaces are calculated using a multivariate linear reconstruction approach [36,37]. In this approach, higher-order accuracy is achieved at the cell faces using the Taylor series of expansion of a cell-centered solution around the cell’s center of gravity [38,39].

(2) $$\phi_{f,SOU}=\phi +\nabla \phi \cdot \vec{r}$$

where ϕ and ∇ϕ are the cell-centered values and its gradient in the opposite cell, and $\vec{r}$ is the vector displacement from the center of gravity of the cell against the direction of the center of gravity of the face.

The given setting was able to deal with the flow with all the changes induced during pumping and fully manage this type of very complex flow and corresponded to the results of experimental measurements [40].

**Figure 4:**
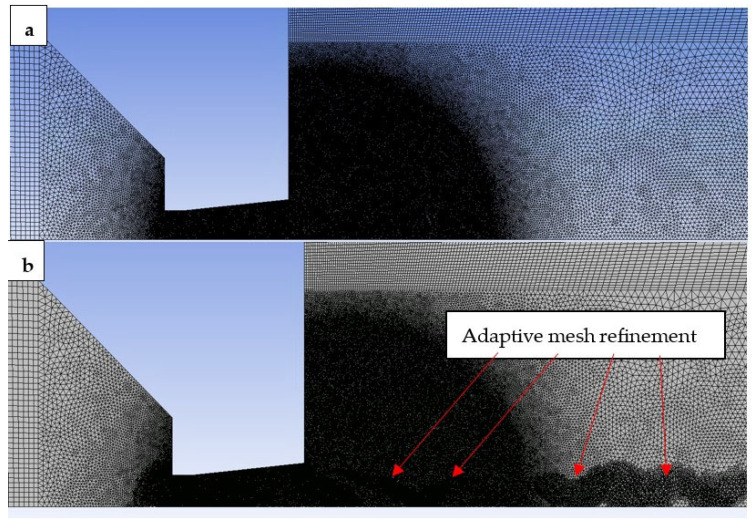
Structured mesh for the mathematical-physics analysis (**a**) basic setting of the mesh, and (**b**) pressure gradients in the supersonic flow regions in the nozzle.

A suitably selected mesh was needed for the chosen mathematical-physics analysis, and the resulting computational mesh is a combination of a structured mesh with a 2D variant of hexagonal elements. The advantage of these meshes is that they conserve cells when meshing purely rectangular surfaces (Figure 2) and also reduce blurred results caused by possible errors in transferring results over oblique ones. A structured mesh cannot be used in the aperture and nozzle areas, so a triangular mesh has been used here. For the most accurate simulation, a significant refinement of the mesh was used in the area where supersonic flow is expected. Figure 4a shows the basic setting of the mesh with refinement in the area of expected more complex physical phenomena in flow and gradients [41]. During the calculation, manual adaptive refinement was also performed using the Field Variable method. The choice extent of mesh adaptation was chosen according to the maximum values in the cell derivative option, a gradient pressure, with a maximum refinement level of 4. As a result, pressure gradients in the supersonic flow regions in the nozzle were appropriately captured, as can be seen in Figure 4b. A mesh independence study was carried out for the inspection, which consisted of monitoring the course of the monitored variables after mesh refinement. Global parameters were monitored: absolute pressure, static temperature, velocity, and density.

**Figure 5:**
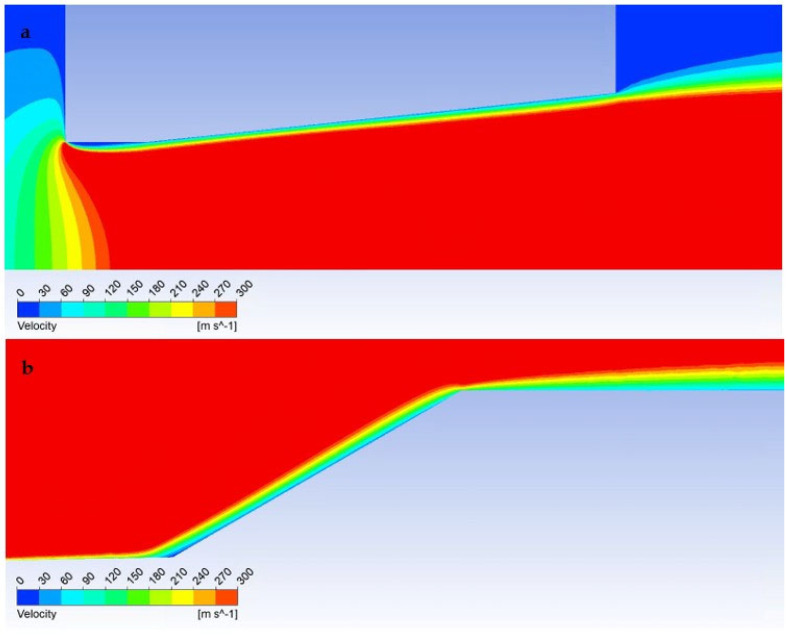
The velocity profile in the boundary layer (**a**) in the nozzle and (**b**) at the tip of the sensor.

An important factor was the setting of the boundary layer. To set it, there is a rule of creating at least 10 cells in the cross-section of the channel from the axis to the wall with refinement at the wall, and in the case of a flowing body, at least five fine cells on the flow around the body. When setting up a turbulent model, the size of the first cell is usually determined using the y+ variable. This option is not available for the laminar model set up by us because there is no turbulent flow and all possible eddies created, for example, by detachment at the edges of the nozzle, are laminar in character without mixing the individual layers of flow. The correctness of the boundary layer settings can then be checked by evaluating the boundary layer, where the so-called velocity profile must be created, as shown in Figure 5.

## 4. Theoretical Materials

### 4.1. Determination of the Computational Cross-Section

For the planned experiments, the calculation state of the nozzle was determined using the theory of one-dimensional isentropic flow for the construction of this nozzle [42]. Thus, according to the dimension of the input cross-section of the nozzle—the narrowest cross-section, the so-called critical cross-section, and the output cross-section of the nozzle was determined [43,44].

This calculation is based on the following relationships of the theory of one-dimensional isentropic flow, which relate state quantities such as pressure, temperature, density, velocity, Mach number, and nozzle flow (Equations (3)–(8)) [45,46].

(3) $$\frac{v_{v}}{v_{kr}}={\left[\frac{\left(\varkappa +1\right)M^{2}}{2+\left(\varkappa -1\right)M^{2}}\right]}^{\frac{1}{2}},$$

(4) $$\frac{v_{v}}{v_{o}}={\left[\frac{2}{2+\left(\varkappa -1\right)M^{2}}\right]}^{\frac{1}{2}},$$

(5) $$\frac{T_{v}}{T_{o}}=\frac{2}{2+\left(\varkappa -1\right)M^{2}},$$

(6) $$\frac{p_{v}}{p_{o}}={\left[\frac{2}{2+\left(\varkappa -1\right)M^{2}}\right]}^{\frac{\varkappa }{\varkappa -1}},$$

(7) $$\frac{\rho_{v}}{\rho_{o}}={\left[\frac{2}{2+\left(\varkappa -1\right)M^{2}}\right]}^{\frac{1}{\varkappa -1}},$$

(8) $$\frac{\rho_{v}}{\rho_{kr}}=\frac{A_{kr}}{A}=M{\left[\frac{\varkappa +1}{2+\left(\varkappa -1\right)M^{2}}\right]}^{\frac{1}{2}\frac{\varkappa +1}{\varkappa -1}},$$

where *p_0_* is the input pressure, *p_v_* is the output pressure, *T_0_* is the input temperature, *T_v_* is the output temperature, *v_0_* is the input velocity, *v_v_* is the output velocity, *v_kr_* is the critical velocity, *ρ_0_* is the input density, *ρ_v_* is the output density, *M* is the Mach number, ϰ is the gas constant for used Nitrogen = 1.4, *A* is the computational cross-section, and *A_kr_* is the critical cross-section.

The nozzle has been designed for the ratio of output to input pressure *P_v_:P_o_* = 0.1.

**Figure 6:**
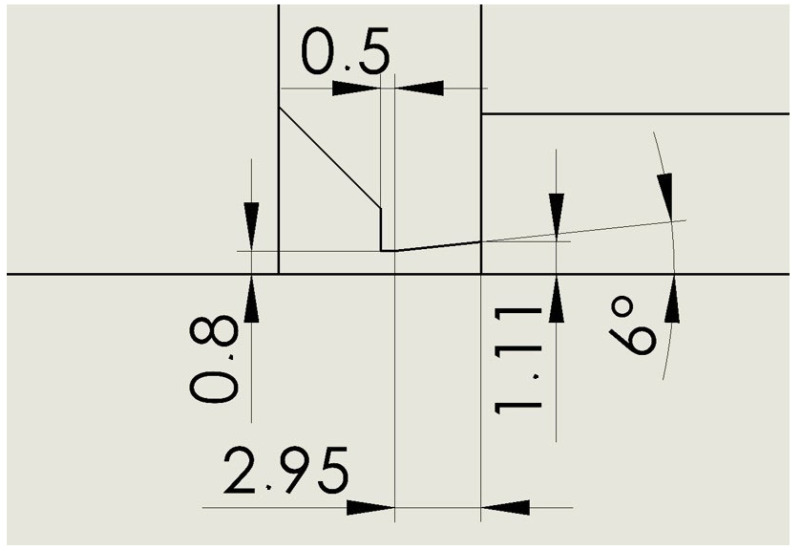
Dimensions of the designed nozzle [mm].

In the case of the designed nozzle, from the above relationships of the theory of isentropic one-dimensional flow (Equations (3)–(8)) based on the input critical cross-section of the nozzle with a diameter of 1.6 mm, an output cross-section with a diameter of 2.22 mm with a length of 0.5 mm and an opening angle of 12° according to [27] was set (Figure 6).

This designed nozzle was analyzed in the Ansys Fluent system. For the calculation, the variant *P_vs_ =* 200 Pa:* P_o_ =* 2000 Pa was chosen.

The boundary conditions setting for mathematical-physics analysis is shown in Figure 2 above.

### 4.2. Gas Flow Regimes

Temperature sensing in the supersonic flow regime is closely related to the flow velocity. The solved problem, which is also reflected in the sensing of temperature in supersonic flow, needs to be described in connection with the sensing of the flow velocity and thus also with static and total pressure.

**Figure 7:**
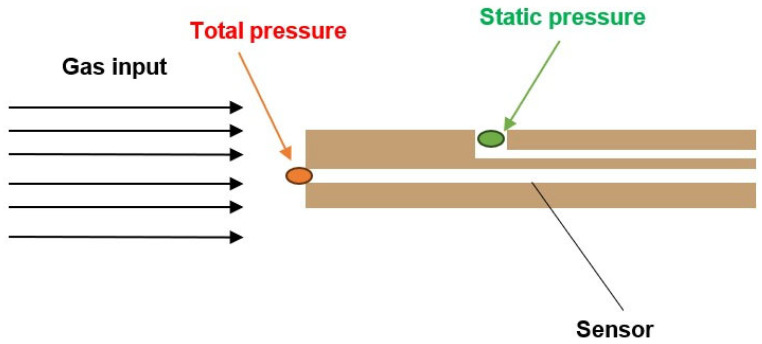
Pitot’s tube sensing method.

As mentioned, one of the options for measuring flow velocity is to use a Pitot tube sensor (Figure 7). This principle is based on the relation where the total pressure is equal to the sum of the dynamic and static pressures.

(9) $$p_{c}=\frac{1}{2}v^{2}+p_{s}$$

Dynamic pressure is further calculated as the product of density and the square of velocity. If we obtain the total pressure from the head of the Pitot tube and the static pressure from its side, the velocity can be determined based on this relationship (Equation (9)). However, it is not always possible to determine it using this simple calculation due to the three different flow regimes, as shown below.

In practice, however, this simple principle of diagnosis can be used only for cases of velocity up to 0.3 Mach, as there are three mathematical flow regimes of solving velocity by Pitot tube, namely [47,48,49]:

1. Incompressible Regime
2. Subsonic Compressible Regime
3. Supersonic Compressible Regime

#### 4.2.1. Incompressible Regime

A flux can be considered incompressible if its velocity is less than 30% of the speed of sound. For such a fluid, Bernoulli’s equation describes the relationship between velocity and pressure along the flow plane, and the simple equation above applies (Equation (10)).

After deriving, it is possible to obtain velocity from the following relationship:

(10) $$v=\sqrt{\frac{2\left(P_{t}-P_{s}\right)}{\rho }}$$

#### 4.2.2. Subsonic Compressible Regime

For flow velocities greater than 30% of the speed of sound, the fluid is considered compressible. In the theory of compressible flow, it is necessary to consider the dimensionless Mach number *M*, which is defined as the ratio of the flow velocity *v* to the speed of sound *c*:

(11) $$M=\frac{v}{c}$$

**Figure 8:**
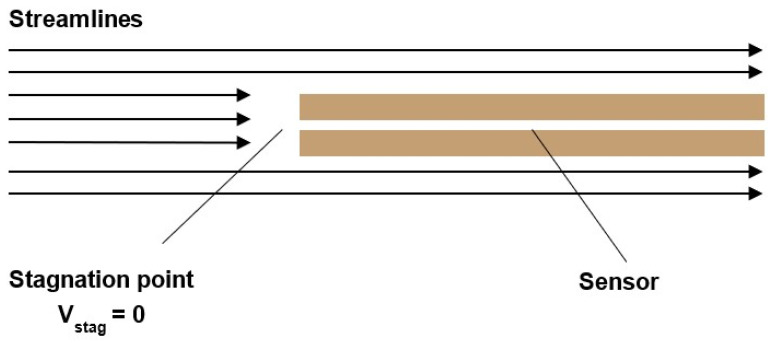
Subsonic compressible regime.

When the Pitot tube is subjected to a subsonic compressible flow rate (0.3 < *M* < 1), the flow of gas along the streamlines ends in smooth compression at the stagnation point of the Pitot tube (Figure 8).

The relationship for determining the velocity takes on a more complex form, which takes into account Poisson’s constant and the value of the stagnation pressure, which is taken by the Pitot tube instead of the total pressure.

(12) $$v=\sqrt{\frac{2\varkappa }{\varkappa -1}\frac{p_{static}}{{}_{static}}\left[{\left(\frac{p_{stagnation}}{p_{static}}\right)}^{\left(\frac{\varkappa -1}{\varkappa }\right)}-1\right]}$$

(13) $$\varkappa =\frac{C_{p}}{C_{v}}=\frac{c_{p}}{c_{v}}$$

where ϰ is the Poisson constant, *C_p_* is the heat capacity at constant pressure, *C_v_* is the heat capacity at a constant volume, and *c_v_* and *c_p_* are the respective specific heat capacities.

#### 4.2.3. Supersonic Compressible Regime

**Figure 9:**
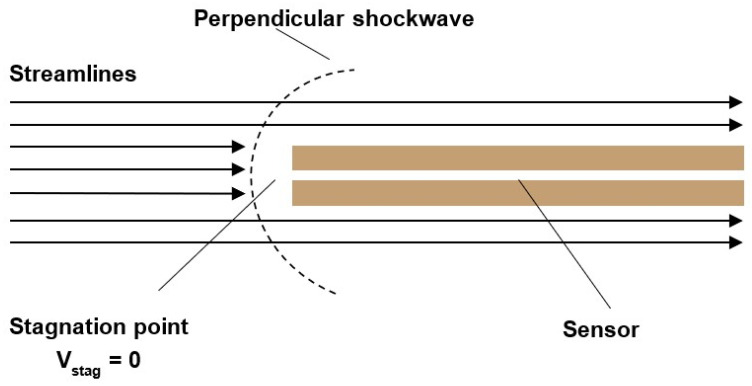
Supersonic compressible regime.

In supersonic mode (*M* > 1), a shockwave is formed in front of the Pitot tube head. The gas is first slowed down non-isentropically to subsonic velocity and then slows isentropically to zero velocity at the stagnation point (Figure 9).

The relationship for determining the velocity can no longer have a common form, but it is the ratio of stagnation pressure sensed from the Pitot tube head and static pressure sensed again from its side. This ratio is expressed by the following relationship:

(14) $$\frac{p_{stagnation}}{p_{static}}=\frac{{\left[\frac{\varkappa +1}{2}M^{2}\right]}^{\left(\frac{\varkappa }{\varkappa -1}\right)}}{{\left[\frac{2\varkappa }{\varkappa +1}M^{2}-\frac{\varkappa -1}{\varkappa +1}\right]}^{\left(\frac{1}{\varkappa -1}\right)}}=\frac{\varkappa +1}{2}M^{2}{\left[\frac{{\left(\varkappa +1\right)}^{2}M^{2}}{4\varkappa M^{2}-2\left(\varkappa -1\right)}\right]}^{\left(\frac{1}{\varkappa -1}\right)}$$

From Equation (14), it is then necessary to express the value of the Mach number using the iterative method, from which we then obtain the value of the flow velocity.

As will be evident from the results of the analysis in the Ansys Fluent system, a large part of the flow behind the aperture moves in this regime.

### 4.3. Temperature Measurement

**Figure 10:**
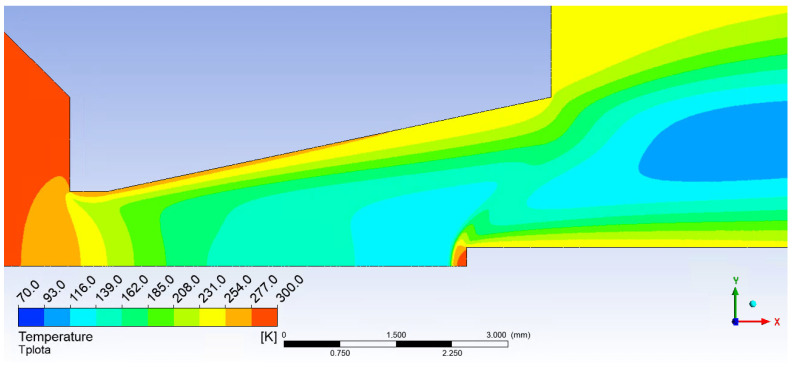
Temperature distribution.

Here, a similar problem with the shockwave manifests itself as with the total pressure sensing. In front of the temperature sensor, the flow is slowed down, and a perpendicular detached shock wave is formed, resulting in an increase in temperature (Figure 10). The temperature sensor senses the stagnant temperature *T*_0_, but the value sought is the static temperature, which is obtained from Equation (15) [50,51]:

(15) $$\frac{T}{T_{0}}={\left(1+\frac{\varkappa -1}{2}M^{2}\right)}^{-1}$$

where *T* is static temperature, *T*_0_ is stagnation temperature, ϰ is Poisson constant, and *M* is Mach number.

### 4.4. Perpendicular—Detached Shock Wave

In the case of a perpendicular detached shock wave, there are sharp gradients behind it, and the temperature, density, and both total and static pressure change abruptly. For this case, there is a theory of one-dimensional isentropic flow for a perpendicular shock wave, which relates the Mach number, velocity, temperature, density, and both total and static pressure that enter and exit the shock wave (Equations (16)–(21)) [46].

(16) $$M_{2}^{2}=\frac{2+\left(\varkappa -1\right)M_{1}^{2}}{2\varkappa M_{1}^{2}-\left(\varkappa -1\right)}$$

(17) $$\frac{T_{2}}{T_{1}}=1+\frac{2\left(\varkappa -1\right)}{{\left(\varkappa +1\right)}^{2}}\cdot \frac{1+\varkappa M_{1}^{2}}{M_{1}^{2}}\cdot \left(M_{1}^{2}-1\right)$$

(18) $$\frac{p_{2}}{p_{1}}=1+\frac{2\varkappa }{\varkappa +1}\left(M_{1}^{2}-1\right)$$

(19) $$\frac{\rho_{2}}{{}_{1}}=\frac{V_{1}}{V_{2}}=\frac{\left(\varkappa +1\right)M_{1}^{2}}{2+\left(\varkappa -1\right)M_{1}^{2}}$$

(20) $$\frac{p_{02}}{p_{01}}={\left[1+\frac{2\varkappa }{\varkappa +1}\left(M_{1}^{2}-1\right)\right]}^{-\frac{1}{\varkappa -1}}{\left[\frac{\left(\varkappa +1\right)M_{1}^{2}}{2+\left(\varkappa -1\right)M_{1}^{2}}\right]}^{\frac{\varkappa }{\varkappa -1}}$$

(21) $$\frac{p_{02}}{p_{1}}={\left[1+\frac{2\varkappa }{\varkappa +1}\left(M_{1}^{2}-1\right)\right]}^{-\frac{1}{\varkappa -1}}{\left[\frac{\varkappa +1}{2}M_{1}^{2}\right]}^{\frac{\varkappa }{\varkappa -1}}$$

where *M* is the Mach number, *V* is the velocity, *T* is the temperature, *P* is the static pressure, *P_o_* is the total pressure, and *ρ* is the density.

### 4.5. Cone Shock Wave

**Figure 11:**
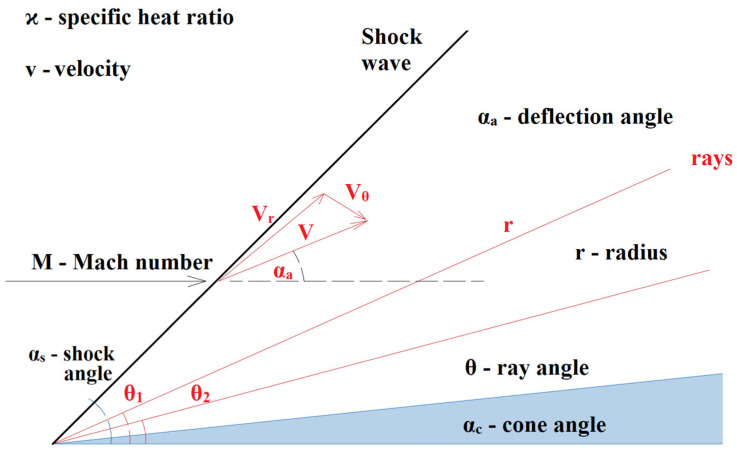
Taylor Maccoll theory.

Since we will be dealing with the cone on the sensor, which ensures the formation of a cone shockwave, it is necessary to briefly mention the problem. The cone shockwave does not show such large changes in state variables, and thus such a large pressure loss happens as a perpendicular-detached shockwave [52]. Due to the conical shape of the sensor inserted into the flow, it is necessary to solve the relationship between the cone and the shockwave according to the Taylor—Maccoll theory (Figure 11) [53].

(22) $$\frac{\varkappa -1}{2}\left[1-v_{r}^{2}-{\left(\frac{dv_{r}}{d\theta }\right)}^{2}\right]\left[2v_{r}+cot\theta \frac{dv_{r}}{d\theta }+\frac{d^{2}v_{r}}{d\theta^{2}}\right]-\frac{dv_{r}}{d\theta }\left[v_{r}\frac{dv_{r}}{d\theta }+\frac{dv_{r}}{d\theta }\frac{d^{2}v_{r}}{d\theta^{2}}\right]=0$$

where *ϰ* is specific heat ratio, *v* is velocity, *M* is Mach number, *s* is shock angle, *a* is deflection angle, *r* is radius, *θ* is ray angle, and *c* is cone angle.

**Figure 12:**
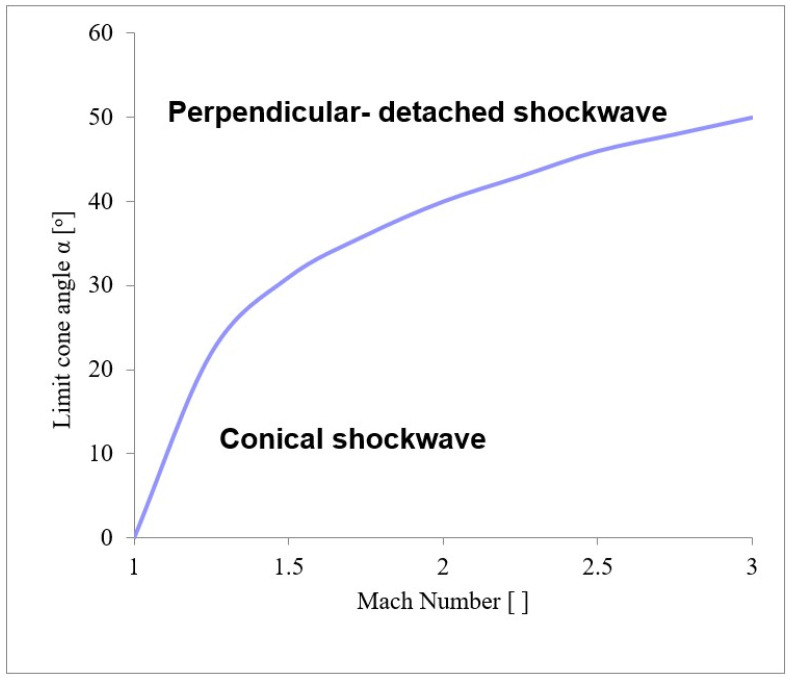
Dependence of Mach number on probe cone angle.

This dependence is possible to plot into the graphics clearly illustrating the Mach number and tip angle values at which the cone shockwave detaches from the sensor tip, resulting in the formation of the perpendicular shockwave (Figure 12) [54,55,56].

The dependence of the angle of the probe cone (α_c_) on the angle of the shock wave (α_s_) is based on Equation (23), where the values for one of the expected measured points in the experimental chamber were entered as input parameters, in which the value of the Mach number *M*_1_ = 2.58 and the selected angle *α_c_* 18° were entered:

(23) $$tg\alpha_{c}=\frac{2{\left(M_{1}\sin\alpha_{s}\right)}^{2}-2}{\left(\varkappa +1\right)M_{1}^{2}-2{\left(M_{1}\sin\alpha_{s}\right)}^{2}+2}ctg\alpha_{s}$$

The change in the values of density, pressure, temperature, and Mach number after passing through the shock wave is also solved by the theory of isentropic one-dimensional flow for a cone shock wave, using a given angle [46].

(24) $$M_{1n}=M_{1}\sin\alpha_{s}$$

(25) $$M_{2}^{2}=\frac{2+\left(\varkappa -1\right)M_{1n}^{2}}{2\varkappa M_{1n}^{2}-\left(\varkappa -1\right)}$$

(26) $$\frac{T_{2}}{T_{1}}=1+\frac{2\left(\varkappa -1\right)}{{\left(\varkappa +1\right)}^{2}}\cdot \frac{1+\varkappa M_{1n}^{2}}{M_{1n}^{2}}\cdot \left(M_{1n}^{2}-1\right)$$

(27) $$\frac{p_{2}}{p_{1}}=1+\frac{2\varkappa }{\varkappa +1}\left(M_{1n}^{2}-1\right)$$

(28) $$\frac{\rho_{2}}{{}_{1}}=\frac{V_{1}}{V_{2}}=\frac{\left(\varkappa +1\right)M_{1n}^{2}}{2+\left(\varkappa -1\right)M_{1n}^{2}}$$

(29) $$\frac{p_{02}}{p_{01}}={\left[1+\frac{2\varkappa }{\varkappa +1}\left(M_{1n}^{2}-1\right)\right]}^{-\frac{1}{\varkappa -1}}{\left[\frac{\left(\varkappa +1\right)M_{1n}^{2}}{2+\left(\varkappa -1\right)M_{1n}^{2}}\right]}^{\frac{\varkappa }{\varkappa -1}}$$

(30) $$\frac{p_{02}}{p_{1}}={\left[1+\frac{2\varkappa }{\varkappa +1}\left(M_{1n}^{2}-1\right)\right]}^{-\frac{1}{\varkappa -1}}{\left[\frac{\varkappa +1}{2}M_{1n}^{2}\right]}^{\frac{\varkappa }{\varkappa -1}}$$

where *M*_1*n*_ is the normal component of Mach number, *M*_2_ is the Mach number behind the cone shock wave, *T*_2_ is the temperature behind the shockwave, *T*_1_ is the temperature in front of the shockwave, *p*_2_ is the static pressure behind the shockwave, *p*_1_ is the static pressure in front of the shock wave, *ρ*_2_ is the density behind the shock wave, *ρ*_1_ is the density in front of the shockwave, *p*_02_ is the total pressure behind the shock wave, and *p*_01_ is the total pressure in front of the shock wave.

## 5. Results

Mathematical-physics analyses of the supersonic flow of the nozzle were performed and compared.

**Figure 13:**
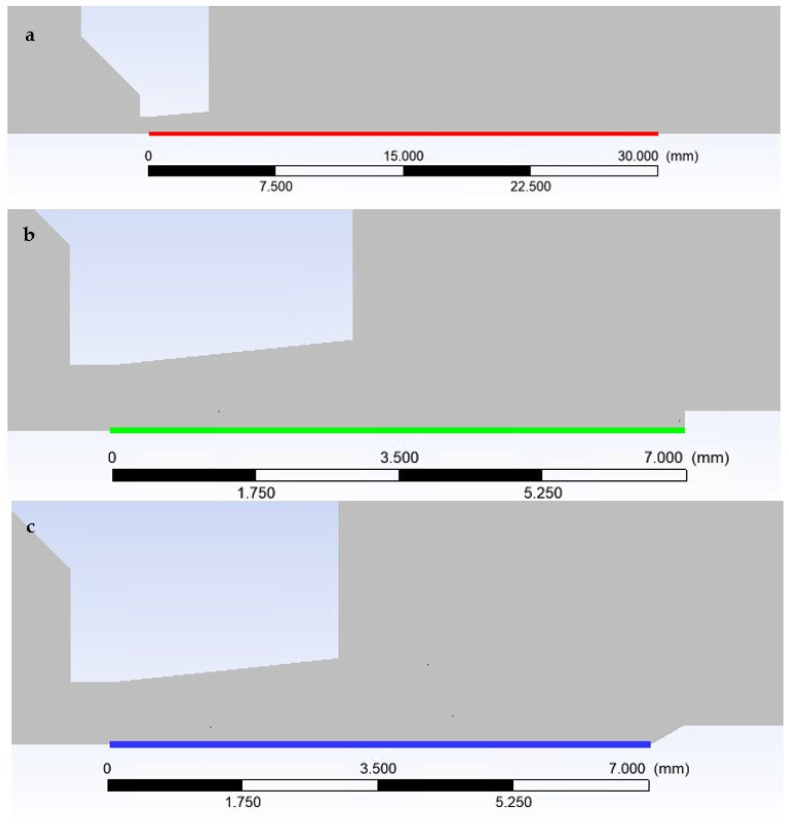
Nozzle without inserted sensor—Free flow (**a**), nozzle with inserted flat-ended sensor—Flat shape (**b**), and nozzle with inserted cone sensor—Angle 30° (**c**).

In the first step, a mathematical-physics analysis was performed on the given shape of the nozzle (Figure 6) without the inserted sensor in the graphics marked as free flow (Figure 13a). In the second step, the analysis was performed with an inserted sensor with a flat end marked as the flat shape in graphics (Figure 13b). In the third step, the analysis was performed with an inserted probe with a conical end with an angle of 30° in graphics marked as Angle 30° (Figure 13c). This color coding is also observed for plotted graphics.

In the following sub-chapters, the individual results will be analyzed and described in the following style: in each sub-chapter, an analysis of the atmospheric pressure variant (pressure ratios between *P_o_* = 101,325 Pa and output *P_v_* = 10,132 Pa) will be carried out first, followed by a comparison with the low-pressure variant (pressure ratios between *P_o_* = 10,000 Pa and output *P_v_* = 1000 Pa), both in a pressure ratio of 10:1, as is shown in Figure 2. Each sub-chapter will have two images for a convenient comparison, where “a” will stand for the atmospheric pressure variants and “b” for the low-pressure variants.

### 5.1. Evaluation of Flow Velocity

Before we proceed to evaluate the average of the main investigated variable in this paper—static temperature—we will evaluate the state quantities related to this and dependent on them. First, the velocity is evaluated in the form of a Mach number.

First, we compare the results of the atmospheric pressure variant.

**Figure 14:**
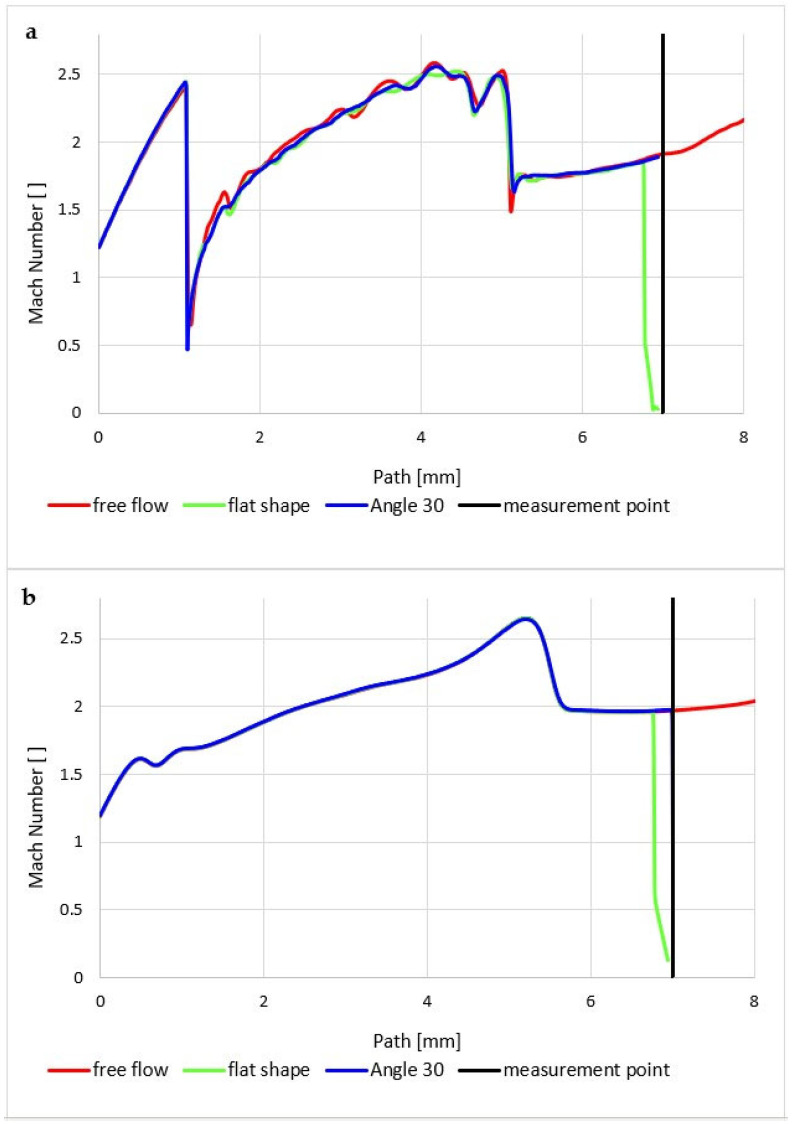
Mach number course of (**a**) atmospheric pressure variant and (**b**) low-pressure variant.

**Figure 15:**
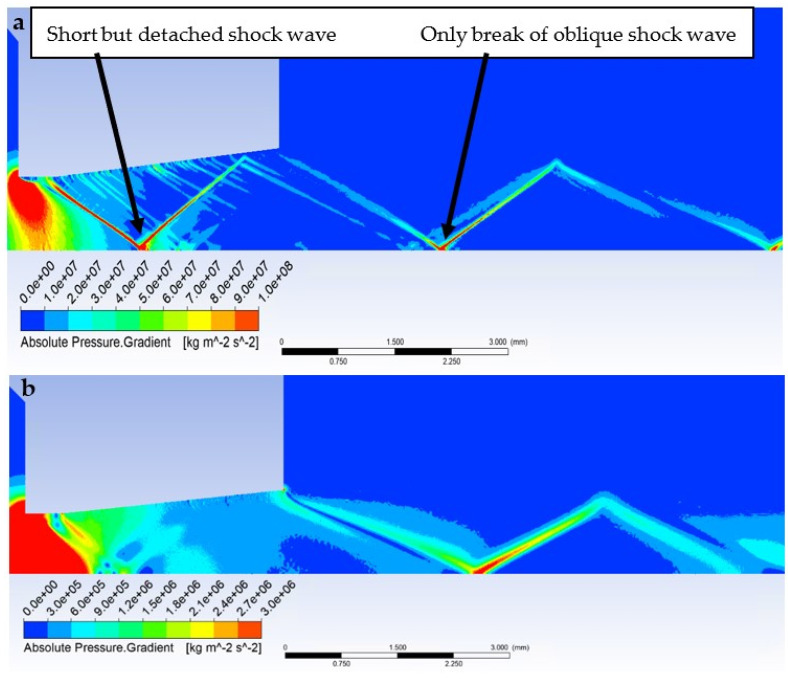
Distribution of Pressure Gradient of Free flow of (**a**) atmospheric pressure variant and (**b**) low-pressure variant.

In the graphics (Figure 14a), a sharp increase can be seen in the Mach number behind the aperture in the first part of the nozzle, up to a distance of 1.2 mm, to a value of almost 2.5 Mach. Then, under the influence of a perpendicular shock wave (Figure 15a), there is a sharp drop in velocity to a subsonic value.

In Figure 15a, this detached shock wave is barely noticeable. It originates from the break of the oblique shock waves. Then, the velocity increases again, and at a distance of about 5 mm it decreases again, but not to subsonic velocity, because this time the gas flow does not pass through a detached but oblique shock wave. Figure 15a also shows that from the break of the oblique shock waves, a detached shock wave, even a short one, does not originate, but only the reflection and break of the oblique shock waves occur. These do not cause a drop in subsonic velocity or sharp gradients of variables such as pressure or temperature, which will be evident in other results.

What is crucial for this case is that in the case of free flow from the passage of the oblique shock wave, there is again an increase in velocity to the next place when the oblique shock wave passes through the axis of the flow, which is evident in Figure 15a in the very right corner at the bottom, which is already outside the range of the distribution, as this is no longer essential for our evaluation.

**Figure 16:**
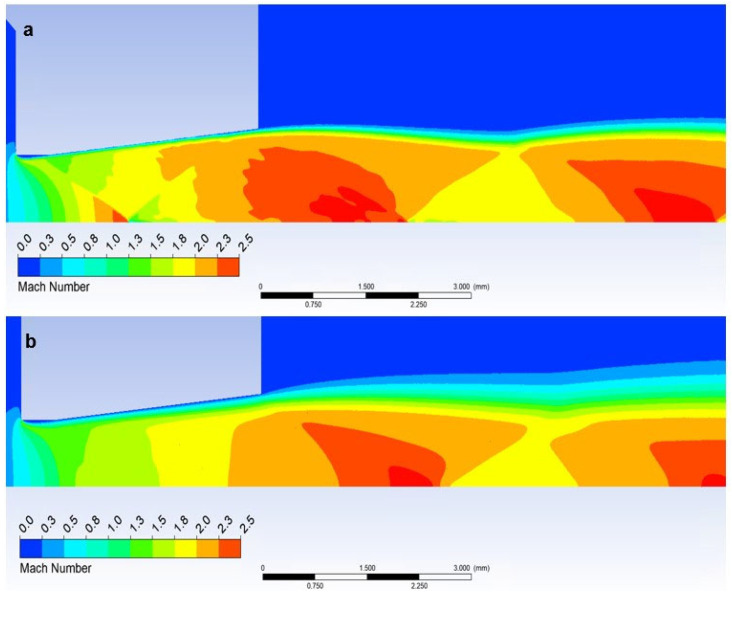
Distribution of Mach number of Free flow of (**a**) atmospheric pressure variant and (**b**) low-pressure variant.

In Figure 16a, the Mach Number distribution shows where the oblique shock waves pass because they change their velocity rapidly and, as will be seen later in the analysis of pressure and temperature, these quantities as well. However, their change is not nearly as significant as the transition on the axis at the point where the short perpendicular shock wave passes.

However, the low-pressure variant is different (Figure 14b); there is no formation of significant oblique shock waves in the nozzle, and no perpendicular shock wave is created (Figure 15b and Figure 16b). Therefore, there are no steep changes to subsonic velocity and the whole process is smoother. This is because the ratio of inertial and viscous forces is different at low pressure. Due to the low pressure (and thus the density), the inertial forces are lower than in the case of the atmospheric pressure variant. However, in the second case, a ratio involving low pressures has been selected, albeit still above the threshold where viscous forces remain unaffected by decreasing pressure. Consequently, viscous forces play a more significant role in this ratio.

A comparison can be made by the quantity of the Reynolds number, which relates inertial and viscous forces, and is the resistance of the environment due to internal friction. The higher the Reynolds number, the lower the influence of the frictional forces of the fluid particles on the total resistance. Internal friction is dependent on the velocity gradient (Equation (31)).

(31) $$\tau =\eta \frac{dv}{dy}$$

where τ is the tangential stress, η is the dynamic viscosity, and $\frac{dv}{dy}$ is the velocity gradient.

Kinematic viscosity is related to dynamic viscosity and is determined by Equation (32).

(32) $$\bar{v}=\frac{\eta }{\rho }$$

where ϱ is density.

The Reynolds number *Re* is given by

(33) $$Re=\frac{vr}{\bar{v}}$$

where *v* is the velocity of the flowing fluid, *r* is the radius of the tube, through which the fluid flows, and $\bar{v}$ is the kinematic viscosity. This relationship can be used in our case in aperture and nozzle.

For fluid flow in spaces of a more general shape than the tube, in our case when flowing around the sensors, the radius of the tube *r* is replaced by a suitable characteristic dimension *l*. Then, applies:

(34) $$Re=\frac{vl}{\bar{v}}$$

**Figure 17:**
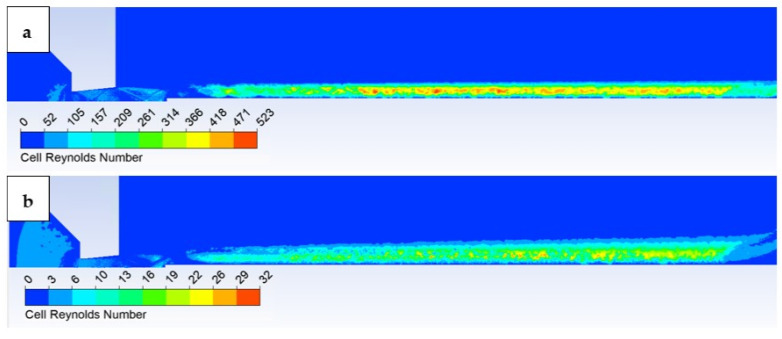
Comparison of Cell Reynolds number (**a**) atmospheric pressure variant, and (**b**) low-pressure variant.

For a basic comparison, we can use the function (local avg. mixture density × local average cell velocity ×cell dimension)/local viscosity in the Ansys Fluent system, where the characteristic dimension is taken according to the size of the Cell Reynolds number (Figure 17).

It is evident that in the low-pressure variant, the influence of frictional viscous forces is significantly smaller than inertial forces, on average up to 1:15.

Now, we will focus on the case with an inserted sensor with a flat end in the atmospheric pressure variant (pressure ratios *P_o_* = 101,325 Pa and output *P_v_* = 10,132 Pa).

**Figure 18:**
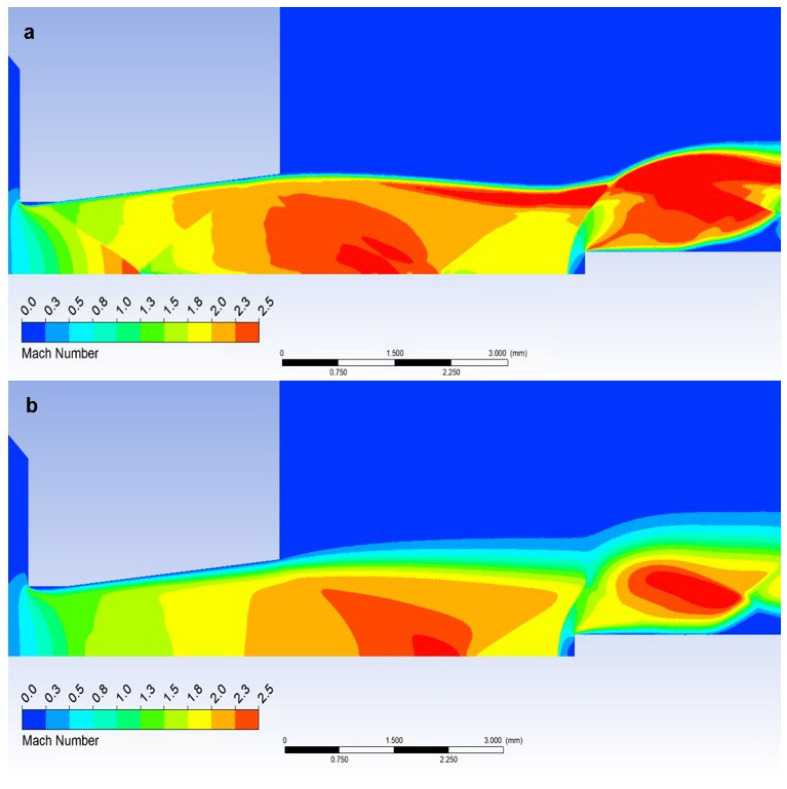
Distribution of the Mach number of Flat shape of (**a**) the atmospheric pressure variant and (**b**) the low-pressure variant.

**Figure 19:**
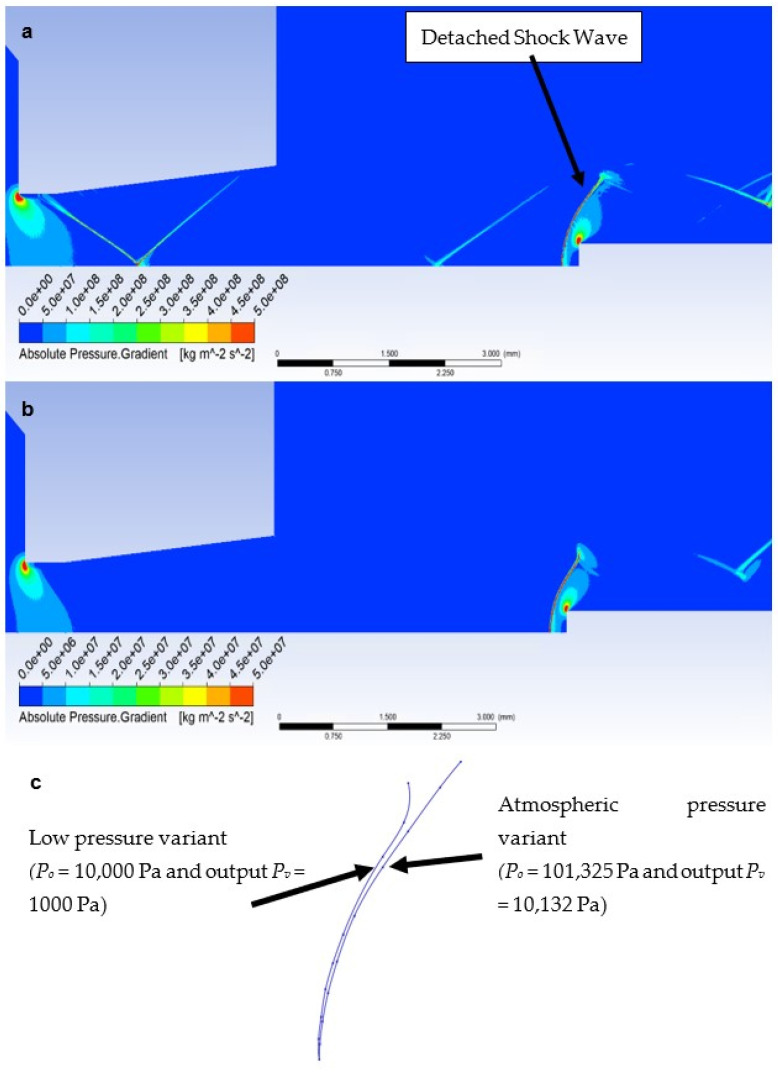
Distribution of Pressure Gradient of Flat shape of (**a**) the atmospheric pressure variant, (**b**) the low-pressure variant, and (**c**) comparison of detached shock waves of both variants.

In our case, however, it is essential that in the variant where we insert a temperature sensor with a flat end into the axis of the flow—Flat Shape, there is a sharp decrease in velocity to stagnation at the surface of the sensor. This can be seen in the graphics in Figure 14a. Figure 18a shows a sharp drop in velocity behind the perpendicular detached shock wave, which is shown in Figure 19a with the pressure gradient. Behind this shock wave occurs the previously mentioned sharp gradient of velocity, pressure, and temperature.

In the figure of the distribution of the Mach number (Figure 18a,b), it is evident that in the atmospheric pressure variant, there are gradients of the Mach number on the oblique shock waves, while these gradients are not present in the low-pressure variant due to the absence of oblique shock waves and the flow has a gradual character.

Due to the different ratios of inertial and viscous forces in the low-pressure variant, i.e., a lower influence of inertial forces, there is a slight change—a smaller rounding of the detached shock wave in the low-pressure variant (Figure 19b). Figure 19c shows a comparison of the detached shock waves of both variants.

**Figure 20:**
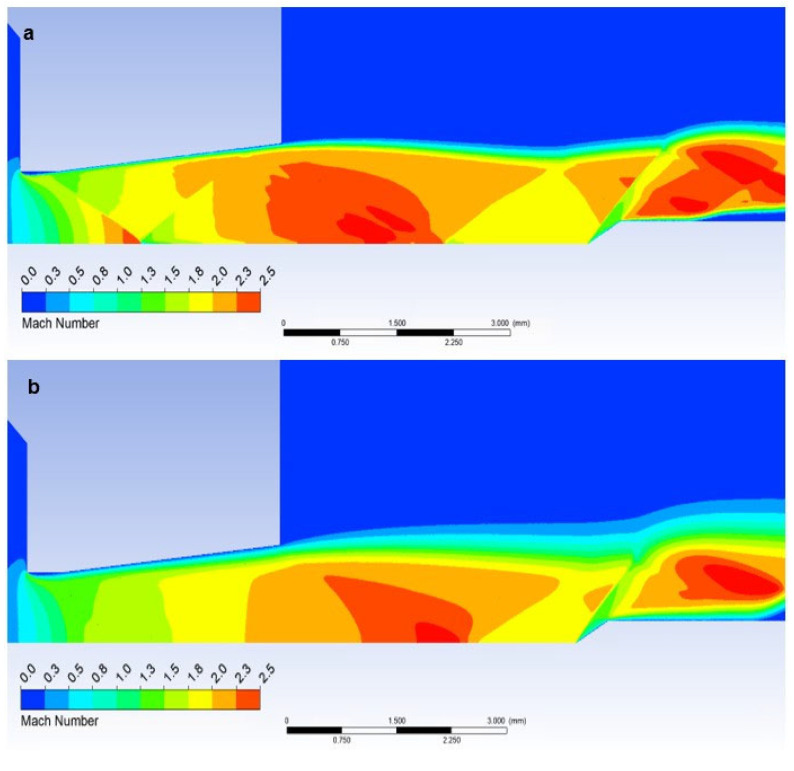
Distribution of Mach number of Angle 30° of (**a**) the atmospheric pressure variant and (**b**) the low-pressure variant.

A completely different process occurs when we insert a temperature sensor with a tip into the flow axis—Angle 30°. Here, there is an incomparably slight drop in speed, but only behind the tip of the sensor tip—behind the cone shock wave. This can be seen from the graph in Figure 14a, which shows that the Mach number waveform is completely identical to the waveform of the variant without the inserted probe and completely different from the Flat Shape variant (Figure 20).

**Figure 21:**
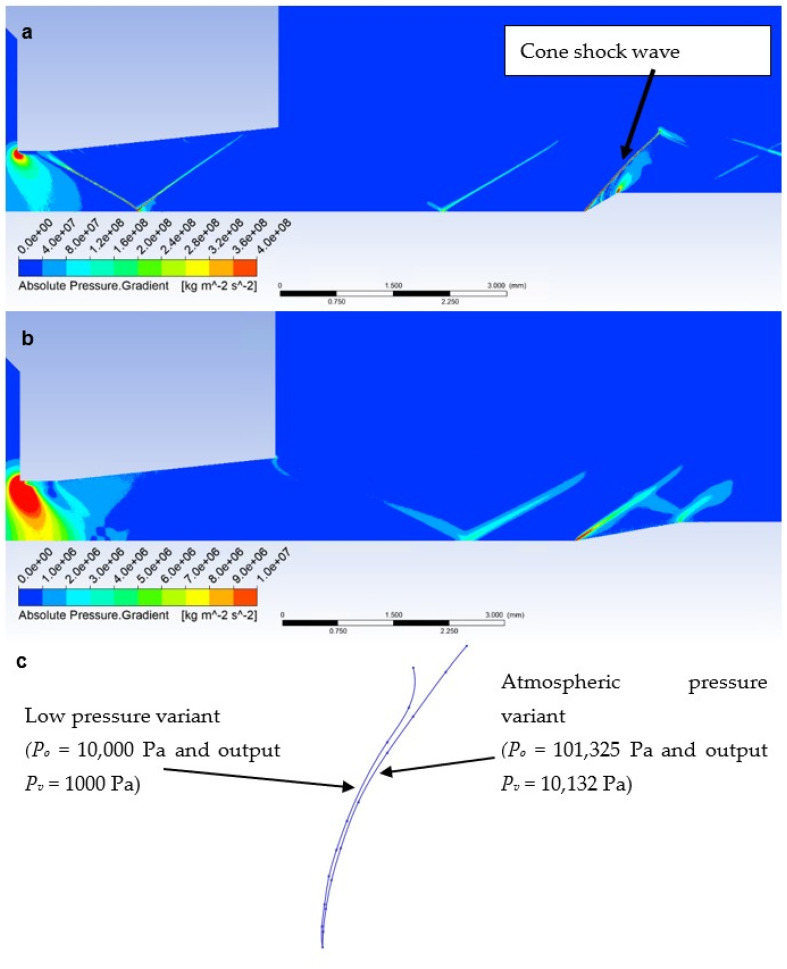
Distribution of Pressure Gradient of Angle 30° of (**a**) the atmospheric pressure variant, (**b**) the low-pressure variant, and (**c**) the comparison of cone shock waves of both variants.

The cone shockwave, which is shown in Figure 21a, is provided by a correctly selected sensor tip angle, as mentioned in Section 4.5. Since the probe is located at a point 7 mm from the beginning of the nozzle (Figure 13a,b), according to the results of the graphics (Figure 14a) of the Mach Number for the Free Flow variant, the Mach Number value at this point is 1.91 Mach. According to the theory (Section 4.5), the cone shock wave is separated from angle values greater than 39°. Therefore, the value of 30° was chosen with a reserve. That is, sensors capable of creating a cone shock wave up to Mach number values of 1.5 and larger.

According to Taylor MacColl’s theory for the velocity Mach Number = 1.9 and a Cone angle of 30°, the cone shockwave angle is 49.8°. This corresponds exactly to the mathematical-physics analysis in the Ansys Fluent system variant in Figure 21a for the atmospheric pressure variant. It is interesting to compare it with the low-pressure variant (Figure 21b). Due to the different nature of the flow, according to mathematical-physics analyses, the velocity at the point of the sensor tip in the free flow is higher than in the previous variant, i.e., Mach Number = 1.97 (Figure 20b). For this velocity and Cone angle of 30°, according to Tayor Maccoll’s theory, the angle of the cone shockwave is smaller than in the previous variant, i.e., 48.6°. In fact, in this variant, a larger angle was measured—52.8° (Figure 21b), so it is a big change. An important observation is that the thickness of the shock wave in the low-pressure variant is also about 0.02 mm thicker due to the smaller impact of inertial forces compared to the constant impact of viscous forces, which will further affect the evaluation of the pressure and temperature curve of the inserted sensor with an angle of 30° (Figure 21c).

### 5.2. Evaluation of Static Pressure

With the help of the discussed results of the Mach number distribution, it is possible to analyze the results of the static pressure waveform.

**Figure 22:**
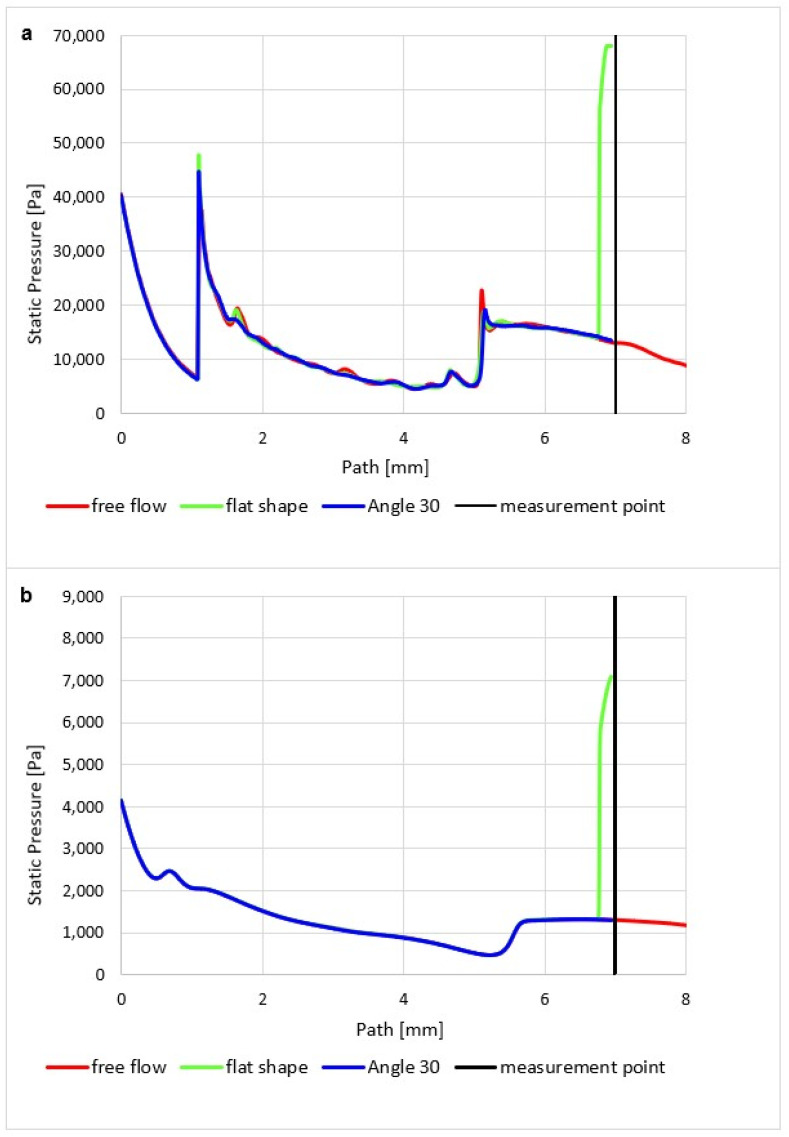
Static pressure course of (**a**) atmospheric pressure variant and (**b**) low-pressure variant.

In the graphics (Figure 22a) for the atmospheric pressure variant, a sharp decrease in static pressure is first noticeable, copying a sharp increase in the Mach number. Then, there is a sharp increase in pressure due to the decrease in velocity to a subsonic value due to the perpendicular shock wave (Figure 21a).

As can be seen in the graphics (Figure 22a), in the case of free flow, another passage occurs, this time through the crossing of oblique shock waves at a distance of about 5.2 mm. There is a slight increase in pressure, and then a decrease again due to an increase in the flow velocity.

**Figure 23:**
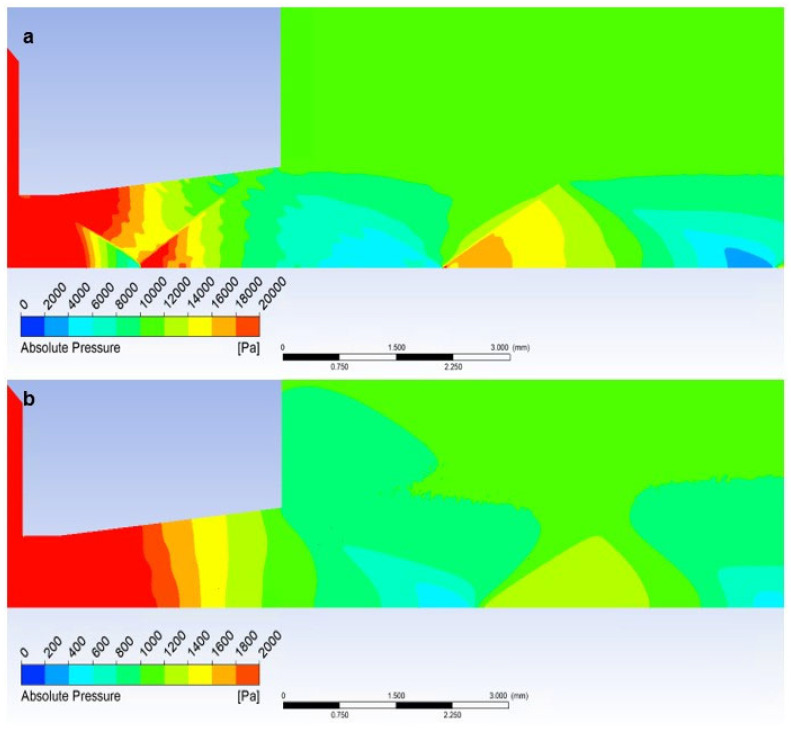
Distribution of Absolute Pressure (Static pressure) of Free flow of (**a**) atmospheric pressure variant and (**b**) low-pressure variant.

Figure 23a clearly shows the pressure distribution with all pressure gradients at the shock wave boundaries. A sharp gradient on the perpendicular shock wave and smaller gradients on the border of oblique shock waves can be seen.

On the other hand, in the case of the low-pressure variant, the pressure curve, which appears gradual without step changes, corresponds to the fact that there are no oblique shock waves, and thus the course of the Mach number does not show large gradients (Figure 23b).

**Figure 24:**
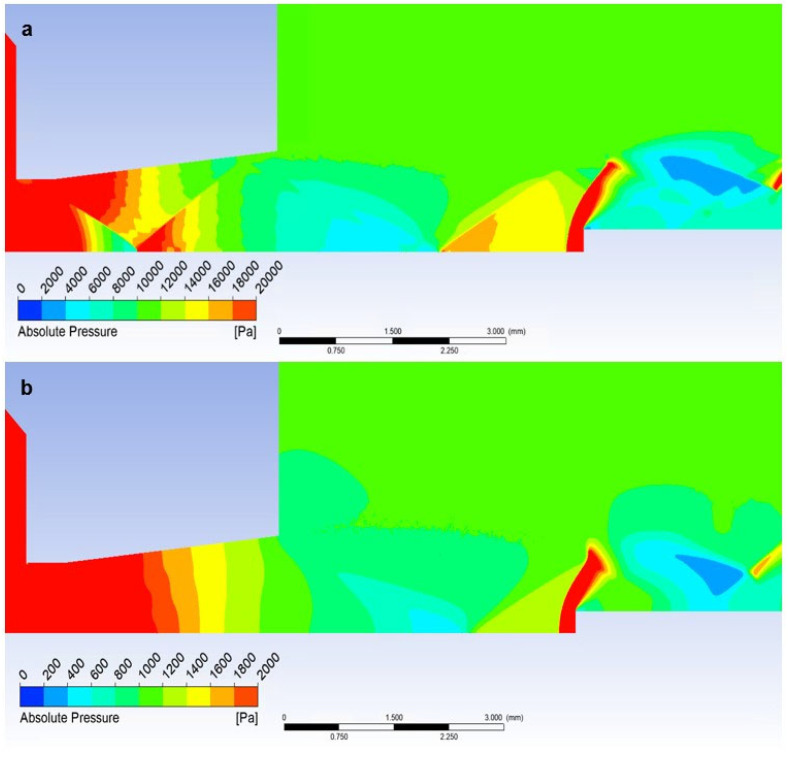
Distribution of Absolute Pressure (Static pressure) of Flat Shape of (**a**) the atmospheric pressure variant and (**b**) the low-pressure variant.

If a temperature sensor with a flat end is inserted into the flow axis, there is a sharp increase in pressure on the sensor surface up to the moment of stagnation. The pressure on the sensor surface is no longer static, but total pressure. This is how the total pressure in the Pitot tube is sensed in the flow. This can be seen in the graphics in Figure 23a. Figure 24a shows a sharp rise in pressure behind the perpendicular detached shock wave, which is shown in Figure 19a using a pressure gradient. Behind this shock wave, there is a sharp increase in pressure since the shock wave is approximately 0.2 mm away from the sensor surface; this area is visible in Figure 24a.

In the case of the atmospheric pressure variant, the total pressure is slightly lower, and as in the case of the low-pressure variant, the flow velocity is higher at the given location, as mentioned in the previous subchapter (Figure 24b).

**Figure 25:**
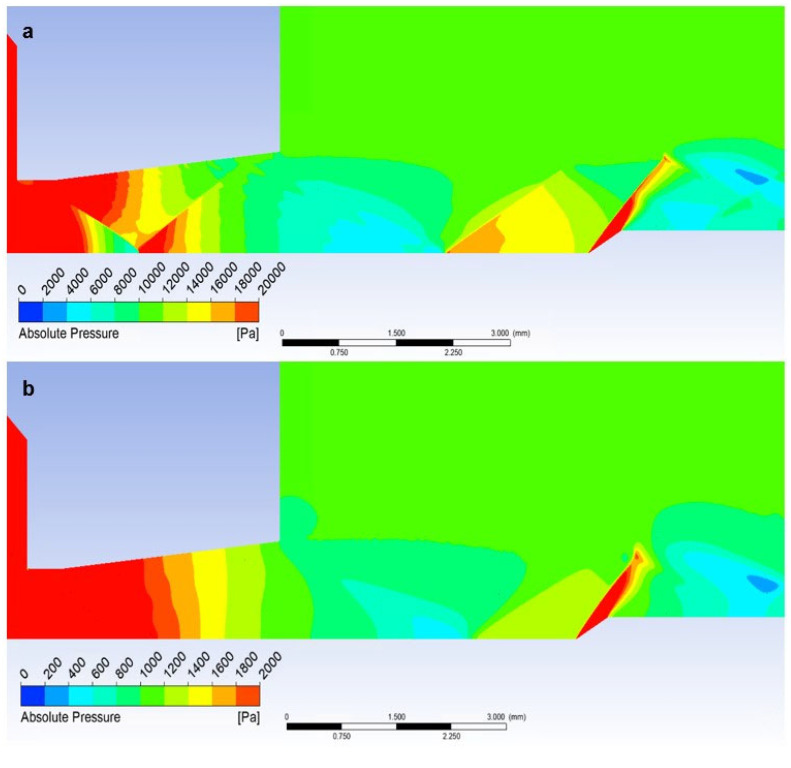
Distribution of Absolute Pressure (Static pressure) of an angle of 30° of (**a**) atmospheric pressure variant and (**b**) low-pressure variant.

Since the state variables are bound, a completely different process occurs when we insert a temperature sensor with the already mentioned tip—an angle of 30°—into the flow axis. Here, there is a remarkably small increase in the pressure value, but what is crucial is that it occurs not in front of the sensor but behind the tip of the sensor—behind the cone shockwave. This can be seen from the graphics in Figure 22a. The static pressure waveform is completely identical to the variant without the inserted probe and completely different from the Flat Shape variant (Figure 25a).

However, the effect of the difference in the ratio of inertial and viscous forces in both variants is manifested. Due to the greater thickness of the shock wave, there is a small increase in static pressure just before the tip, which is not visible in the graphics because it is at a distance of 0.007 mm (Figure 21b and Figure 22b). This must be considered when using a temperature sensor with a tip, as this small increase in static pressure is also reflected in an increase in static temperature (Figure 25b).

### 5.3. Evaluation of Static Temperature

The course of static temperature is dependent on the course of Mach number and Static pressure and is very similar to the course of static pressure.

**Figure 26:**
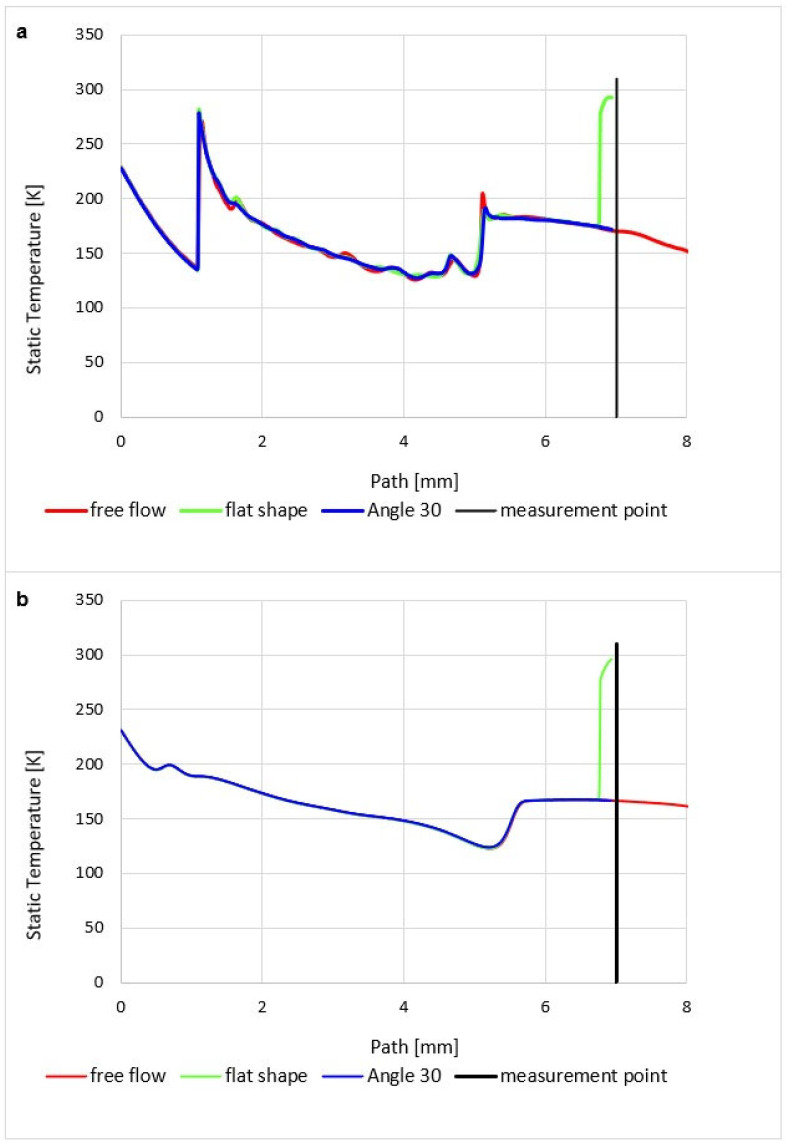
Static temperature course of (**a**) atmospheric pressure variant and (**b**) low-pressure variant.

The graphics (Figure 26a) show a sharp decrease in static temperature following a pressure drop and, conversely, a sharp increase in Mach number. Again, there are gradients caused by perpendicular and oblique shock waves (Figure 15a).

The temperature distribution in the Free Flow variant corresponds to the stated characteristics of the supersonic flow in the nozzle.

In the case when a temperature sensor with a flat end is inserted into the flow axis, the temperature of the sensor surface behind the detached shock wave (Figure 19a) rises sharply until it reaches the stagnation temperature value on the nozzle surface (Equation (35)). Therefore, the temperature on the sensor surface is no longer static, but stagnant, corresponding to the total temperature. Its difference is evident in the graph (Figure 26a), which shows the difference between static and stagnation temperatures at the point of the inserted sensor–measurement point.

The analysis is carried out according to the theory given in Section 4.3. From the results in the graphics for the course of Mach number = 1.91 (Figure 14a), static temperature *T =* 171 K (Figure 26a), and Poisson’s constant for Nitrogen ϰ = 1.4, it is possible to determine the stagnation temperature *To* from the modified relationship,

(35) $$T_{o}=\frac{T}{{\left(1+\frac{\varkappa -1}{2}M^{2}\right)}^{-1}}=303K$$

which in the currently examined case amounts to up to 123.5 K and corresponds to the value obtained by mathematical-physics analysis in Figure 29a.

**Figure 27:**
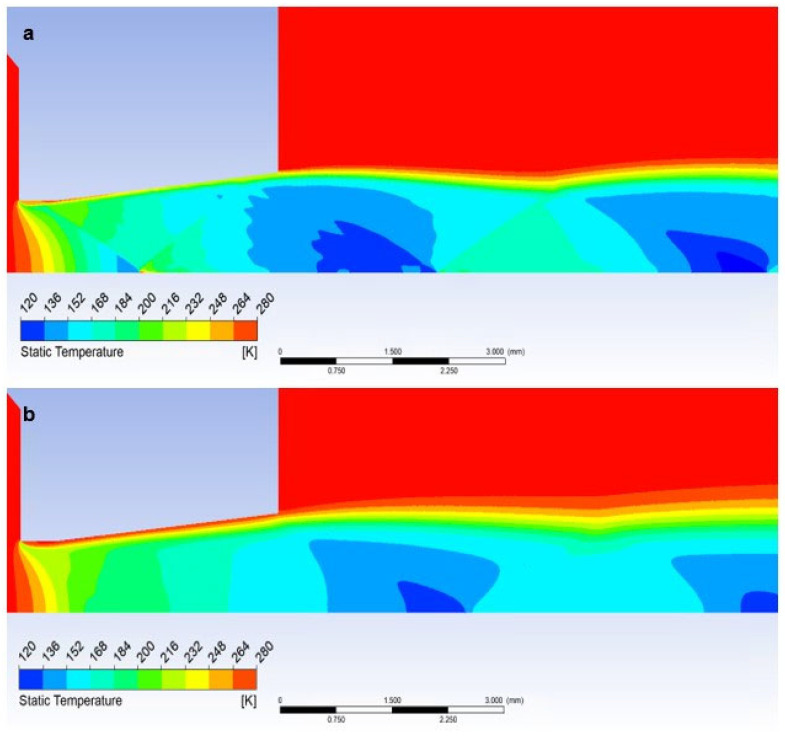
Distribution of Static temperature of Free flow of (**a**) the atmospheric pressure variant and (**b**) the low-pressure variant.

**Figure 28:**
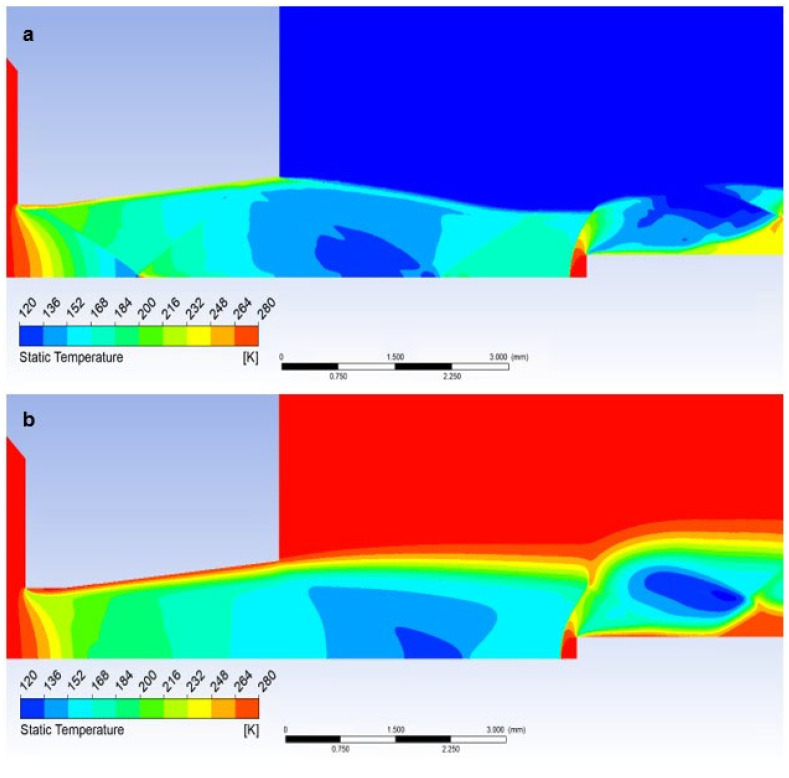
Distribution of Static temperature of Flat shape of (**a**) the atmospheric pressure variant and (**b**) the low-pressure variant.

Here, as in the course of static pressure, the graphics (Figure 26a) and Figure 27a show a sharp rise in pressure behind the perpendicular detached shock wave, which is shown in Figure 15a, using a pressure gradient. Behind this shock wave, there is a sharp increase in pressure and temperature. In Figure 28a, this area is marked by a distinctive red area.

**Figure 29:**
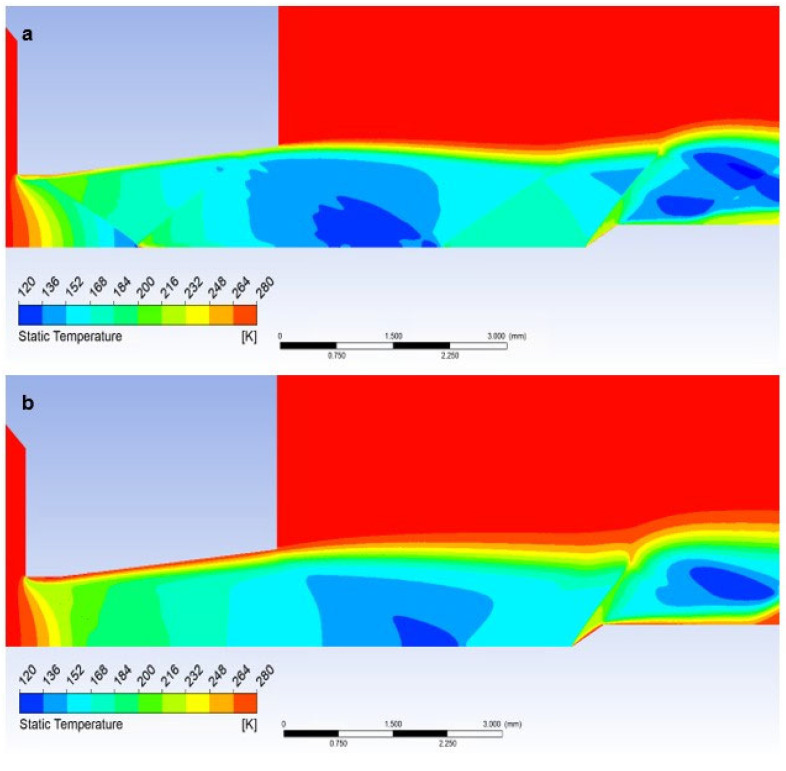
Distribution of Static temperature of Angle 30° of (**a**) atmospheric pressure variant and (**b**) low-pressure variant.

Inserting a sensor with a 30° tip into the gas flow causes the same effect as in the previous case when analyzing the static pressure waveform. Even in this case, there is a remarkably small temperature rise, but what is crucial, it occurs behind the tip of the sensor tip—behind the cone shock wave. This can be seen from the graph in Figure 26a, where the static temperature curve is completely identical to the course of the variant without an inserted probe and completely different from the Flat Shape variant (Figure 29a).

This fact is of great importance for static temperature scanning using a conventional sensor with a flat end—Flat Shape. As previously mentioned, the sensor measures stagnation temperature, and to derive static temperature, it becomes imperative to ascertain the Mach number. If it is determined by scanning, for example, with a Pitot tube, it is an extra operation and it is also an introduction of an error into the measurement.

When using a suitably shaped sensor tip, we directly sense the actual static temperature, and the value of the Mach number can be obtained from the pressures in front of the nozzle and behind the nozzle according to the relationships (Equations (23)–(30)). To determine the angle of the tip, it is sufficient to assume that the tip angle can be selected with a sufficient reserve.

A small problem may arise with the low-pressure variant, where the different influence of the ratio of inertial and viscous forces in both variants takes place. Due to the greater thickness of the shockwave, a small increase in static pressure happens just before the tip, which is difficult to see in the graph as it is a distance of 0.007 mm (Figure 21b,c and Figure 26b). It has a value of up to 1500 Pa. This must be taken into account when using a temperature sensor with a tip, as this small increase in static pressure is also reflected in a static temperature increase of 78 K (Figure 28b and Figure 29b). In this case, it would be necessary to create the sensor tip with a significantly sharper tip angle, otherwise, the results would be distorted. When sensing the temperature, it is necessary to take into account the different shapes of the shockwave at low pressure.

This paper serves as the basis for a planned experiment with the Schlieren method, in which it will be placed in the experimental chamber using the windows visible in Figure 1. The principle of the planned Schlieren method is based on the bending of the light beam trajectory when passing through an inhomogeneous transparent object. Unlike the Shadowgraph method, a filter (optical knife) is used, which is implemented by inserting an aperture into the focal length of the imaging lens. The resulting image of the Schlieren method represents the first derivation of the density of the screened medium. Due to its simplicity and clarity, the Schlieren method is used to visualize heat transfer, momentum, or flow of matter and will be used for experimental verification of the results of mathematical and physical analyses from Ansys Fluent.

## 6. Conclusions

In this paper, a mathematical-physics analysis was carried out in conjunction with theoretical data in the field of supersonic flow behind the nozzle with an inserted temperature sensor. After the presentation of the theoretical background for the issue of flowing the inserted probe into the supersonic flow, including the theory of shockwaves, comparative analyses of inserted probes with both a flat end and a tip for the analysis of the formation of detached and cone shock waves were performed. These analyses were carried out in two variants for pressure ratios of 10:1 before and after the nozzle. The first variant was used for the pressure in the chamber in front of the nozzle in the value of atmospheric pressure. These analyses for the atmospheric pressure variant also served to a certain extent to fine-tune the system, as they also corresponded to theoretical assumptions. The second type of analysis was performed for the low-pressure variant. This choice was deliberately chosen because it is already a low-pressure region, which is a research topic for the team, but it is still an area of continuum mechanics, and it is also intentionally a pressure area, where the viscosity value is still not dependent on pressure. In these variants, the character of the flow was investigated at the ratio of inertial and viscous flow forces for atmospheric pressure conditions, which were compared with low-pressure conditions, where the ratio of inertial and viscous flow forces already leads to a significant reduction in inertial forces.

The results showed a changed character of the flow with a reduced tendency towards the formation of cross-oblique shock waves in the nozzle and the formation of shock waves with a greater thickness because the viscous forces further inhibited the formation of the thickening of the shockwave itself. This can affect temperature sensing with a sensor with a tip, as the shockwave area can form in a very small area in front of the tip, which can affect the result. Also, due to the reduced inertial forces, the angle of the cone shock wave is a few degrees larger than in theory; there is no such tilting due to the lower inertial forces.

These analyses are the basis for the upcoming experiments in an experimental chamber built for experiments in the given region of low pressures on the boundary of continuum mechanics.

## Data Availability

The data presented in this study are available on request from the corresponding author.
